# Neutrophil in Reverse Migration: Role in Sepsis

**DOI:** 10.3389/fimmu.2021.656039

**Published:** 2021-03-15

**Authors:** Jingjing Ji, Jie Fan

**Affiliations:** ^1^Department of Surgery, University of Pittsburgh School of Medicine, Pittsburgh, PA, United States; ^2^Department of Critical Care Medicine, General Hospital of Southern Theater Command of PLA, Guangzhou, China; ^3^Research and Development, Veterans Affairs Pittsburgh Healthcare System, Pittsburgh, PA, United States; ^4^McGowan Institute for Regenerative Medicine, University of Pittsburgh, Pittsburgh, PA, United States

**Keywords:** PMN, sepsis, inflammation, rerverse migration, infection

## Abstract

Sepsis is life-threatening organ dysfunction caused by a dysregulated host response to infection. During the development and progression of sepsis, polymorphonuclear neutrophils (PMNs) are the most abundantly recruited innate immune cells at sites of infection, playing critical roles in the elimination of local infection and healing of the injury. PMN reverse migration (rM) describes the phenomenon in which PMNs migrate away from the inflammatory site back into the vasculature following the initial PMN infiltration. The functional role of PMN rM within inflammatory scenarios requires further exploration. Current evidence suggests that depending on the context, PMN rM can be both a protective response, by facilitating an efficient resolution to innate immune reaction, and also a tissue-damaging event. In this review, we provide an overview of current advancements in understanding the mechanism and roles of PMN rM in inflammation and sepsis. A comprehensive understanding of PMN rM may allow for the development of novel prophylactic and therapeutic strategies for sepsis.

## Introduction

Polymorphonuclear neutrophils (PMNs) are the first responders in the circulation, playing an important role in defending against invading pathogens (1). Being attracted by a chemokine concentration gradient, PMNs migrate from the bloodstream into the inflamed extravascular tissues (2, 3). In the inflammatory site, PMNs eliminate pathogen through degranulation, phagocytosis, the formation of neutrophil extracellular traps (NETs), and releasing cytokines (3, 4). After PMNs execute their antimicrobial agenda, timely clearance of PMNs is crucial to maintain homeostasis (2, 5, 6). Traditionally, apoptosis or necrosis and subsequent phagocytosis by macrophages were considered as the main ways for PMN clearance (7, 8). However, with the development of imaging technology, it has been found that the recruited PMNs could migrate back to the circulation, which serves as a new way of PMN clearance in inflammatory or injury site (8, 9). In 1997, Hughes et al. using a rat glomerular capillary injury model and radiolabeling of PMN found that over 70% of PMNs that entered inflamed glomerular capillaries were able to return to the main circulation without undergoing apoptosis in the inflammatory site (8, 10). The process of PMN migrating back to circulation has been referred to as “PMN reverse migration (rM)” to describe the general phenomenon of PMN moving in the opposite direction to that expected (11). The *in vivo* visualization of PMN rM was first carried out on zebrafish larvae in 2006 (12). Mathias et al. found that not all recruited PMNs died at the site of injury, and most PMNs left the site and back to circulation (13). In the same year, Buckley et al. observed *in vitro* that human PMNs could reverse transmigrate through the tumor necrosis factor-α (TNF-α)-activated endothelial monolayer (14). Further studies showed that reverse migrated PMNs are characterized by high expression of intercellular adhesion molecule-1 (ICAM-1 high) and low expression of C-X-C motif chemokine receptor 1 (CXCR1 low), which are different from the PMN in circulation with ICAM-1 ^low^/CXCR1 ^high^ or those resident in tissue with ICAM-1 ^high^/CXCR1 ^high^ (15, 16). The markers of ICAM-1 ^high^/CXCR1 ^low^ are then found also valid for the reverse migrated PMNs in the peripheral blood of patients with systemic inflammation (14). In 2017, *in vivo* PMN rM in mice was visualized by Wang et al. in a sterile thermal hepatic injury model, in which reverse migrated PMNs from the inflammatory site were imaged in the lungs and bone marrow (17). The published data support the note that PMN rM is an important biological conservative phenomenon existing in from zebrafish to humans (11, 14, 17–21).

## Current Knowledge on The Mechanisms of PMN rM

The infiltration of PMNs from circulation to an inflammatory site is a regulated multi-stage process, as summarized in several review articles (2, 6, 22). PMNs first recognize inflammatory signs by sensing chemokines, e.g., macrophage-inflammatory protein-2 (MIP-2) and keratinocyte-derived chemokine (KC) (23, 24), followed by several processes, including capture, rolling, firm adhesion, and transendothelial migration, to reach the site of inflammation (25, 26). Corresponding to these steps, PMN rM has been considered as a continuous multi-stage process as well. Sussan et al. proposed terminologies to describe cell reverse migration, including reverse abluminal crawling (rAC), reverse interstitial migration (rIM), reverse luminal crawling (rLC), and reverse transendothelial cell migration (rTEM) (11). These terminologies imply the mechanisms by which PMN rM occurs.

**Figure 1:**
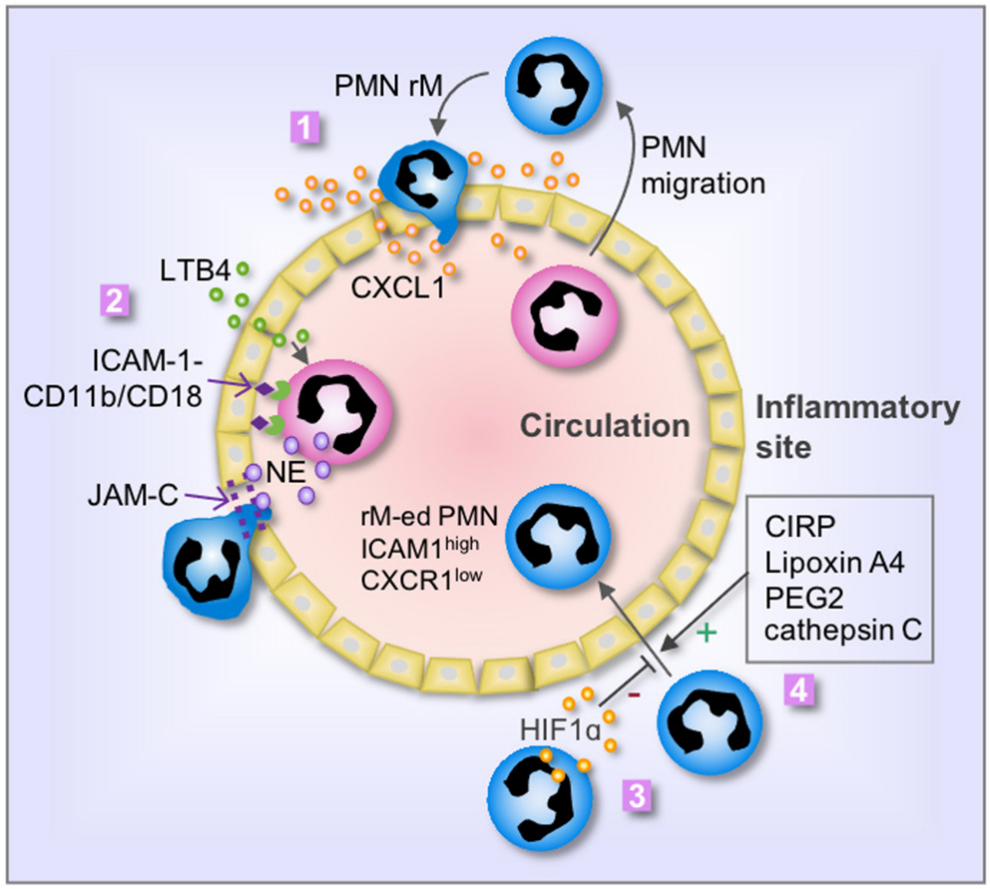
Mechanisms of PMN rM. **1**. Breach of endothelium results in leakage of chemokine, i.e., CXCL1, from the inflammatory site into the vasculature, therefore driving PMN to reenter the circulation; **2**. LTB4 induces PMN release of neutrophil elastase (NE), which in turn cleaves endothelial JAM-C and subsequent damage of endothelial junction and promotes PMN rM. The binding between PMN CD11b/CD18 and endothelial ICAM-1 retains the PMN on the surface of endothelial cells and secures the cleavage of JAM-C by NE; **3**. Activation of HIF1α suppresses PMN rM; and **4**. Many factors, including cold-inducible RNA-binding protein (CIRP), Lipoxin A4, PEG2, and cathepsin C, can promote PMN rM. The phenotype of reverse migrated PMN (rM-ed PMN) is ICAM1^high^ CXCR1^low^.

The mechanisms that mediate PMN rM from inflammatory sites remain largely unclear. Many factors, which involve in PMN forward migration, such as chemoattractant and chemotactic repellents, chemokine receptors, the interaction between PMN and endothelia, and alteration of PMN behavior, are also considered as main factors regulating PMN rM (Figure 1).

During the PMN forward migration, PMNs are chemoattracted by chemokines, and the migration direction is controlled by the concentration gradient of the chemoattractant. Endothelium breach is an important event in the PMN migration from circulation to the inflammatory area (27, 28). Similarly, the alteration of endothelial junction and permeability also play an important role in the mechanism of PMN rM. Owen-Woods et al. revealed the importance of endothelial permeability in regulating PMN trafficking (29). Inflammation usually damages the endothelial junctions and increases endothelial permeability, thereby, resulting in leakage of chemokines from the inflammatory site to circulation. This leakage of chemokines may reverse the directional cues of PMN migration across venular walls (30). For example, the leakage of chemokine and PMN chemoattractant, CXCL1, impairs the chemoattractant gradient and confuses PMNs and droves PMNs reenter the systemic circulation (29, 31). Regarding the mechanism underlying endothelial junction damage, Colom et al. reported that neutrophil elastase (NE) cleaves endothelial junctional adhesion molecule C (JAM-C), therefore, promoting PMN rM (32). This process is mediated by leukotriene B_4_ (LTB4). The study showed that LTB_4_ exhibits high efficacy in promoting rapid NE release and/or cell-surface expression, which then mediates the cleavage of endothelial JAM-C (32). Decreasing LTB4 concentration or blocking LTB4 receptor could decrease PMN rM in a mouse model of acute pancreatitis (33). Hirano et al. found that inhibition of JAM-C degradation reduces PMN rM in septic mice (34). The study further showed that the soluble JAM-C concentration in circulation is positively correlated with the PMN rM, and thus, increased plasma soluble JAM-C level may serve as an indicator for PMN rM occurring (32).

PMN transepithelial migration is an important event during PMN migration. However, the study on the mechanism of reverse PMN transepithelial migration is lacking in the literature. Future studies are needed to explore the mechanism since reverse PMN transepithelial migration is an indispensable piece of the puzzle for a comprehensive understanding of PMN rM.

Studies have shown that some chemokines and chemokine receptors are critically involved in PMN rM. CXCR1 and CXCR2 are the main receptors on PMNs to sense chemokines during cell migration. The study showed that reverse migrated PMNs exhibit decreased CXCR1 (14). Therefore, it was speculated that those PMNs may lose sensitivity to chemokine cues and move in a reverse direction. However, the direct evidence is still missing. Powell et al. reported that CXCL8a and CXCR2 are required for PMN rM in zebrafish, evidenced by that CXCR2 knockout in zebrafish decreased PMN rM (35). Wang et al. reported that the PMNs that reentered into circulation entailed a sojourn in the lungs, where they up-regulated CXCR4 expression followed by homing back to the bone marrow. The PMNs trafficking back to bone marrow exhibit increased Annexin V in the membrane, suggesting that those PMNs may undergo apoptosis in bone marrow (17).

PMN forward migration to the inflammatory site requires an important step of adhesion to endothelium, which is mediated through the binding between PMN Cd18/Cd11b and endothelial ICAM-1. During PMN rM, Colom et al. found that the binding between Cd18/Cd11b and ICAM-1 secures JAM-C cleavage by neutrophil elastase, which subsequently promotes PMN rM (32).

Some studies revealed negative regulation of PMN rM. For example, the role for hypoxia-inducible factor 1 subunit alpha (HIF1α) in suppressing PMN rM in zebrafish has been reported (36). Activation of HIF1α reduced PMN rM and delayed inflammation resolution in zebrafish; whereas, administration of HIF1α inhibitor enhanced PMN rM (36). Besides, Wang et al. found that reducing serine protease activity by using cathepsin C deficient mice led to a profound reduction in PMN rM (17). Considering that PMNs reenter into circulation followed by migration into the lungs and sequentially homing back to bone marrow, PMN rM is more likely a programmed process, rather than a random event.

Chemotactic repellent in the inflammatory site is another mechanistic hypothesis for PMN rM (8). CXCL8 is an effective chemokine in human. It has been found *in vitro* that CXCL8 functions as a chemoattractant in lower concentrations (at the nM level), whereas, in higher concentrations (at the μM level), CXCL8 plays a role as chemorepellent to promote PMN rM (37). Loynes et al. reported that eicosanoid prostaglandin E_2_ (PGE2) can also act as a chemorepellent that drives PMN rM in zebrafish (38). In the late stage of inflammation, pathways of production of pro-inflammatory mediators are usually shifted to the production of pro-resolution mediators, i.e. lipoxin A_4_ (LXA4) (39, 40). It has been found in a microfluidic device that LXA4 enhances human PMN rM, suggesting that PMN rM may serve as a partial mechanism of inflammation resolution (41). Macrophages also play a role in regulating PMN rM (42). It has been observed in zebrafish that macrophage depletion decreased PMN rM and resulted in continuous neutrophilic inflammation (38). Redox-Src family kinase (SFK) signaling in macrophages seems important in mediating macrophage-induced PMN rM, although redox-SFK also involves in regulating PMN forward migration (43).

Collectively, published studies underscore the complexity and diversity of mechanisms driving PMN rM, highlighting the need to better understand PMN rM characteristics, prevalence, and downstream implications.

## Biological Effect of PMN rM

Both PMN infiltration in tissue and timely clearance are important for maintaining homeostasis, as concluded previously (9, 44). Persistent and excessive PMN infiltration is responsible for many chronic diseases, such as pulmonary fibrosis and rheumatoid arthritis, etc. (45, 46). The functional role of PMN rM in inflammatory scenarios is controversial and requires further exploration. Current evidence suggests that depending on the context, PMN rM can be both a protective response, by facilitating an efficient resolution to innate immune reaction, and also a tissue-damaging event (8, 13, 17, 22, 32, 47–49). Removal of activated PMNs from the inflammatory sites through PMN rM may alleviate the local inflammatory response. On the other aspect, however, activated PMNs that migrate back to circulation may result in dissemination of inflammation (32, 47, 50). The different outcomes may be related to the different disease models, severity, and timing.

Nonetheless, clearance of infiltrated PMNs from the injury or infection sites is essential for inflammation resolution. Most studies in zebrafish found that PMN rM plays a protective role in promoting inflammation resolution. Several interventions aiming to inhibit PMN rM, such as macrophage depletion and activation of HIF1α, aggravated the damage of the wound due to the persistent neutrophilic infiltration (36, 38). Similar results have also been observed in a mouse model of sterile thermal hepatic injury. Using photoactivatable-GFP transgenic mice, Wang et al. visualized the migration of PMNs back into the vasculature in a sterile thermal hepatic injury. They found that reducing serine protease activity by using cathepsin C–deficient mice (Ctsc^−/−^) led to a profound reduction in PMNs reentering healthy patent vessels, and further resulted in delayed revascularization. Though the mechanism was unclear, Ctsc deficiency only decreased PMN rM, but did not affect PMN infiltration, suggesting that Ctsc involved in inflammation resolution (17).

On the other aspect, data from mammal experiments showed that activated PMNs migrating back to circulation contributes to inflammation dissemination and distant organ dysfunction. In a murine model of cremaster muscle ischemia-reperfusion, inhibition of PMN rM by knockout NE or JAM-C resulted in the alleviation of the remote lung, heart, and liver injury, suggesting that those reverse migrated PMNs may contribute to the distant organ injury (32, 47). The mechanism of how the reverse migrated PMNs lead to distant organ damage remains unclear. This may relate to the interaction between reverse migrated PMNs and circulating cells. Studies have shown that the PMN subsets, which present similar phenotype with reverse migrated PMNs, modulate T cell functions, as summarized by Hirano et al. (51). PMNs with increased cell surface expression of CD11b, CD11c, CD16, and CD54 demonstrate the ability to suppress T cell proliferation in a Cd18/Cd11b dependent manner (51).

It is noticeable that reverse migrated PMNs have an altered phenotype consisting of increased expression of ICAM-1 and effector functions, e.g., increased ROS generation (14, 47). Expression of ICAM-1 on the surface of inactivated PMNs is usually low. PMN expression of ICAM-1 can be induced by chemokines, and thus, infiltrated PMNs in the site of injury or infection present upregulated expression of ICAM-1 (49, 50). Whether the high expressed ICAM-1 involves in the mechanism of PMN rM is under investigation in the authors' laboratory.

## PMN rM in Sepsis

Sepsis is life-threatening organ dysfunction caused by a dysregulated host response to infection (52). In the progression of sepsis, PMNs are the most abundantly recruited immune cells at sites of infection, playing critical roles in the elimination of local infection and the healing of injury (53–55). Although the mechanisms underlying sepsis development and progression remain to be fully addressed, it is well-accepted that immune imbalance serves as a critical mechanism of the development and progression of sepsis, in which PMNs play important roles (56, 57). PMNs execute their functions to eliminate the pathogen mainly through degranulation, phagocytosis, the formation of neutrophil extracellular traps (NETs), and release of cytokines. These mechanisms, however, may induce tissue damage as well (54, 55).

Theoretically, timely removal of PMNs in a resolution phase from the inflamed site should benefit local inflammation. However, active PMNs that migrate back to systemic circulation may disseminate inflammatory responses to remote organs and tissue. In the development of sepsis, pathogen-associated molecular patterns (PAMPs) and damage-associated molecular patterns (DAMPs) are the early initiators; and PMNs play an important role in further amplifying the inflammation during the progression of sepsis (8, 58). Inflammatory mediators released by PMNs can exaggerate other innate immune cell activation, including PMN itself (8, 59). Reverse migrated PMNs present a pro-inflammatory phenotype, including increased production of superoxide and high membrane expression of ICAM-1 (47, 60). Ode *et al* found that ICAM-1 positive PMNs express higher levels of inducible nitric oxide synthase (iNOS) and NETs, suggesting that reverse migrated PMNs are still highly active (60, 61). In addition, reverse migrated PMNs show prolonged lifespan and delayed apoptosis (62, 63), which might contribute to persistent and amplified inflammation (64). Studies using the mouse abdominal infection model revealed that the ratio of reverse migrated PMNs in circulation shows a positive correlation with acute lung injury (33, 50, 60). A study using the cecal ligation and puncture (CLP) mouse model showed that cold-inducible RNA-binding protein (CIRP) knockout mice exhibited decreased PMN rM and lung injury. After administration of recombinant CIRP, the reverse migrated PMNs significantly increased in the blood in the time- and dose-dependent manner, and NE expression was upregulated, while JAM-C expression was downregulated in the lungs. These results suggest that CIRP promotes PMN rM and subsequent acute lung injury by increasing NE and decreasing JAM-C (50). Interestingly, the CIRP-induced PMN rM occurred in septic mice can be suppressed by neutralizing antibody against TLR4 and inhibitor for NF-κB. In CLP mice, CIRP-TLR4 interaction in PMNs leads to increased PMN rM through the NF-κB pathway. The reverse migrated PMNs produce excessive iNOS and NETs and promote tissue inflammation and injury (60). Li et al. using another sepsis model, the acute pancreatitis model, revealed that LTB4 production promoted PMN rM, and this effect was mediated by substance P. Substance P treatment increase phosphorylation of protein kinase C (PKC) α and mitogen- activated protein kinases (MAPKs), which further promoted LTB4 production. Blocking the leukotriene B4 receptor 1(LTB4R1) resulted in the decreased PMN rM into the circulation and alleviated the severity of acute lung injury (33).

Although the data on the role of PMN rM in sepsis are limited and it is too early to conclude, the published studies tend to suggest a detrimental effect of PMN rM on the development of remote organ injury in sepsis.

## Summary

The mechanism of sepsis remains to be fully elucidated, which results in poor therapeutic outcomes for septic patients. PMNs have become an important target for preventive and therapeutic interventions. Despite advances in understanding PMN biology over the last several decades, there remains a significant gap in our knowledge regarding various PMN functions and behavior in sepsis. PMN rM describes the phenomenon in which PMNs migrate away from the inflammatory site back into the vasculature following the initial PMN infiltration. The functional role of PMN rM within inflammatory scenarios, particularly in sepsis, requires further exploration. Current evidence suggests that depending on the context, PMN rM can be both a protective response, by facilitating an efficient resolution to innate immune reaction, and also a tissue-damaging event through the dissemination of inflammation. The investigation on the mechanism of PMN rM is still in a premature stage. Numerous questions are still open in the research of PMN rM. For example, what are the mediators in the circulation that chemoattract the PMNs leaving from the inflammatory sites? Whether the reverse migrated PMNs are a particular subset of PMNs? What are the different functions between the infiltrating PMNs and reverse migrated PMNs? Where is the final destination and fate of the reverse migrated PMNs? Nonetheless, the potential physiological and pathological roles of PMN rM emphasize the importance of gaining more in-depth insight into these phenomena, since these may serve as novel means of modulating inflammation and treatment of sepsis.

## Author Contributions

JJ collected the data and drafted the manuscript. JJ and JF conceived and designed the study. JF reviewed and finalized the manuscript. Both authors read and approved the final manuscript.

## Conflict of Interest

The authors declare that the research was conducted in the absence of any commercial or financial relationships that could be construed as a potential conflict of interest.

## Abbreviations

CIRP: Cold-inducible RNA-binding protein
CLP: Cecal ligation and puncture
Ctsc: Cathepsin C
CXCL1: C-X-C motif chemokine ligand 1
CXCL8a: Chemokine (C-X-C motif) ligand 8a
CXCR1: C-X-C motif chemokine receptor 1
CXCR2: C-X-C motif chemokine receptor 2
CXCR4: C-X-C motif chemokine receptor 4
DAMPs: Damage associated molecular patterns
HIF1α: Hypoxia inducible factor 1 subunit alpha
ICAM1: Intercellular adhesion molecule-1
iNOS: inducible nitric oxide synthase
JAM-C: Junctional adhesion molecule C
KC: Keratinocyte-derived chemokine
LTB_4_: leukotriene B4
LTB4R1: leukotriene B4 receptor 1
LXA4: lipoxin A4
MAPKs: Mitogen activated protein kinases
MIP-2: Macrophage-inflammatory protein-2
NE: Neutrophil elastase
NETs: Neutrophil Extracellular Traps
NF-κB: Nuclear factor kappa B subunit 1
PAMPs: Pathogen-associated molecular patterns
PGE2: Prostaglandin E2
PKC: Phosphorylation of protein kinase C
PMN: Polymorphonuclear neutrophils
rAC: reverse abluminal crawling
rIM: reverse interstitial migration
rLC: reverse luminal crawling
rM: reverse migration
rTEM: reverse transendothelial-cell migration
SFK: Src family kinase
TLR4: Toll-like receptor 4
TNF-α: Tumor necrosis factor-α.
